# 2-dimensional shear wave elastography: Interobserver agreement and factors related to interobserver discrepancy

**DOI:** 10.1371/journal.pone.0175747

**Published:** 2017-04-17

**Authors:** Kibo Yoon, Woo Kyoung Jeong, Yongsoo Kim, Min Yeong Kim, Tae Yeob Kim, Joo Hyun Sohn

**Affiliations:** 1 Department of Radiology, Hanyang University Guri Hospital, Hanyang University College of Medicine, Guri, Gyeonggi-do, Korea; 2 Department of Radiology and Center for Imaging Science, Samsung Medical Center, Sungkyunkwan University School of Medicine, Seoul, Korea; 3 Department of Internal Medicine, Hanyang University Guri Hospital, Hanyang University College of Medicine, Guri, Gyeonggi-do, Korea; Universite de Nantes, FRANCE

## Abstract

**Purpose:**

To evaluate the interobserver reproducibility of two-dimensional shear wave elastography (2D-SWE) in measuring liver stiffness (LS) and to investigate factors related to liver 2D-SWE.

**Materials and methods:**

A prospective study was conducted between August 2011 and August 2012 in rheumatoid arthritis patients who had been treated with methotrexate. Interobserver reproducibility of 2D-SWE was evaluated, and the relationship between interobserver difference in LS and related factors was analyzed using linear regression analyses. We considered age, sex, alanine transaminase, cholesterol, body mass index (BMI), and waist circumference as clinical factors, and the mean value of standard deviation (SD^M^), its difference between two examiners, mean diameter of the regions of interest (ROI^M^), and its difference in the elasticity map as investigation factors. The cut-off value for significant factors to predict interobserver discrepancies in LS-based fibrosis stage was also inspected.

**Results:**

In total, 176 patients were enrolled. The intraclass correlation coefficient between the two examiners was 0.784. In the univariate analysis, SD^M^ and ROI^M^ were independently associated with interobserver differences in LS as well as BMI, waist circumference, and the difference of ROI, but SD^M^ and ROI^M^ were the only ones significantly related in multivariate analysis (*p*<0.001 and *p =* 0.021, respectively). The best cut-off value for SD^M^ in predicting interobserver discrepancy in LS-based fibrosis stage was 1.4.

**Conclusions:**

Interobserver reproducibility of 2D-SWE for measuring LS was good and SD^M^ was the most significantly associated factor with interobserver differences in LS and interobserver discordance in LS-based fibrosis stage.

## Introduction

The measurement of liver stiffness (LS) using ultrasound (US) elastography, widely used for the estimation of hepatic fibrosis by viral hepatitis, is increasingly being used in patients with hepatic injuries from various causes [1, 2]. Although hepatic fibrosis evaluated by histopathologic examination is currently considered the diagnostic method of choice, US elastography was developed as a non-invasive alternative method to overcome the disadvantages of biopsy, including invasiveness, sampling error, and interpretational variability [3]. Transient elastography is on the frontier of US elastography, and is useful for detection of liver cirrhosis, prediction of variceal development, and evaluation of response to anti-viral treatments [4, 5]. The clinical utility of various types of US elastography has been well documented [6, 7].

Two-dimensional shear wave elastography (2D-SWE), which uses shear wave speed to create an elasticity map [8], has some advantages compared with transient elastography: higher accuracy; integration into conventional ultrasound; developed discrimination using a broad bandwidth; and improved diagnostic efficiency using a real-time quantitative elasticity map [9, 10, 11]. Nevertheless, interobserver agreement of 2D-SWE has not been verified, in contrast to transient elastography. Several studies have focused on the reproducibility of LS using 2D-SWE [12, 13, 14], but the study design had some limitations. First, it included only a small number of subjects and all were healthy volunteers. Second, the experience level of the examiners was too variable (range, novice to expert). Third, their result was not about the subjects who underwent elastography for screening of liver fibrosis. Fourth, they did not consider the reliability parameter of a single measurement such as standard deviation (SD) in the region of interest (ROI) associated to the reliability of 2-D elastography.

To clarify these issues, we designed a study to verify the reproducibility of 2D-SWE in a situation similar to clinical practice, such as screening for hepatic fibrosis in patients with long-term administration of methotrexate for the treatment of rheumatoid arthritis. The aims of this prospective study were to evaluate the interobserver reproducibility of 2D-SWE and to assess the clinical and investigation factors related to reproducibility of 2D-SWE in assessing liver elasticity.

## Materials and methods

### Patients

The present study was a single-center prospective observational study and was approved by the institutional review board of Hanyang University Guri Hospital (2011–039). We studied the prospective cohort of a concurrent study about LS of rheumatoid arthritis patients who had been treated with methotrexate and who provided written informed consent between August 2011 and August 2012 [15]. The inclusion criteria were consecutive patients diagnosed with rheumatoid arthritis, treated with methotrexate. The exclusion criteria were as followings: 2D-SWE examination was not performed by two examiners on the same day, technical failure of 2D-SWE due to artifacts or an unsaturated elasticity map (less than 50% of the color map), lack of patient cooperation, unavailable clinical data, and serum alanine transaminase (ALT) level over 100 U/L, because increased ALT levels suggest the necroinflammation of hepatocytes and may disturb the prediction of fibrosis in the patients treated with methotrexate.

Medical records were reviewed and the following parameters collected for each patient at the time of 2D-SWE examination: age, sex, serum ALT level, serum cholesterol level, body mass index (BMI), and waist circumference.

### Liver stiffness measurement

**Fig 1:**
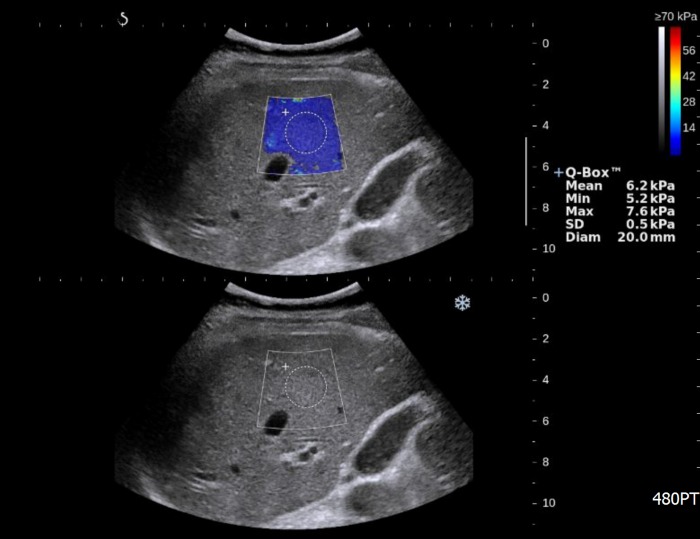
US elastography image for measurement of liver stiffness. The trapezoidal colored box shows the distribution of elasticity in liver tissue, and the round ROI (Q-box) is located to measure elasticity. Measured values are displayed on the right side of the screen: Mean is the mean value of elasticity in the ROI; Min is the minimum elasticity; Max is the maximum elasticity; Std Dev is the standard deviation of elasticity in the ROI; and Diam is the diameter of the ROI.

LS measurement by 2D-SWE was performed following conventional liver US. The patients fasted for approximately eight hours before the examination with an US machine equipped with an SWE module (Aixplorer version 3, Supersonic imagine, Aix-en-Provence, France) and a convex broadband probe (SC6-1). Three staff radiologists (W.K.J., Y.K., and M.Y.K.) alternately performed 2D-SWE in pairs each day. The three examiners had more than 9 years of clinical experience in abdominal radiology and each had used 2D-SWE more than 100 times to measure LS. During expiration, a trapezoidal color elasticity map was positioned on the right liver through the intercostal sonic window to enable a good view of the liver parenchyma, and then the round ROI was placed in the elasticity map to measure the mean value and SD of the elasticity (Fig 1). Generally, we kept the following guidelines for liver stiffness measurement: 1) LS is measured by round ROI when the color map is saturated as large as possible; 2) the depth of measurement ranged 2-5cm from the liver capsule to avoid artifact around liver capsule; 3) the diameter of ROI is 20 mm of dimension, but it is changeable to the situation (e.g. the color map is not fully saturated (due to multiple defective areas). The measurement was repeated five times by each examiner, and all were blinded to the results of the other examiners [16]. The median value of the five repetitions was used to represent LS. We also calculated the mean value of SDs (SD^M^) and mean diameter of the regions of interest (ROI^M^) in the elasticity map to inspect their relationship with LS.

### Statistical analysis

We reviewed the distribution of parametric variables, LS, SD^M^, and ROI^M^ and found that the histograms of these parameters seemed to be skewed left, so these variables were log transformed and validated as having a normal distribution before statistical analysis.

#### Interobserver agreement of LS, SD^M^, and ROI^M^

We calculated the intraclass correlation coefficient (ICC) to evaluate interobserver reproducibility of mean and median LS, SD^M^, and ROI^M^ between the first and second observations [17]. The ICC used a variance component analysis for a two-way mixed effects model without interaction variance [e.g. ICC (3, 1)], which was type A ICC using an absolute agreement definition between the measurements. The following scoring system was used: ICC ≥ 0.87, excellent; 0.87 > ICC ≥ 0.71, good; 0.71 > ICC ≥ 0.50, fair; and ICC < 0.5, poor agreement [18].

#### Factors related to interobserver differences in LS

To explore interobserver differences, we calculated the absolute difference in LS between two consequent observations. SD^M^, ROI^M^, difference in SD^M^ between two observations, difference in ROI^M^ between two observations, and the clinical factors mentioned above were considered as possibly related factors. Univariate linear regression analysis was performed, and variables with a *p* value < 0.20 during univariate analysis were included in multivariate analysis.

#### Factors related to interobserver discrepancies in LS-based fibrosis stage

Liver fibrosis in every patient was staged by two examiners using 2D-SWE on four scales according to the classification system previously suggested by our group: F0-F1 = LS ≤ 8.60 kPa; F2 = 8.60 kPa < LS ≤ 10.46 kPa; F3 = 10.46 kPa < LS ≤ 14.00 kPa; and F4 = LS > 14 kPa [19]. We then investigated which factors among BMI, waist circumference, SD^M^, and ROI^M^ were related to interobserver discrepancies in liver fibrosis using a stepwise multivariate logistic regression analysis, and mean LS was considered as a covariant to correct the size of discrepancy between high and low levels of the LS. Diagnostic performance was evaluated using receiver operating characteristic (ROC) curve analysis. The optimal cut-off was specified by the maximum Youden’s index.

Statistical analyses were performed using SPSS for Windows (version 23; SPSS Inc. Chicago, IL) and MedCalc for Windows (version 14.12.0; MedCalc Software, Mariakerke, Belgium). A *p* value of < 0.05 was considered to be statistically significant.

## Results

### Enrolled subjects

**Fig 2:**
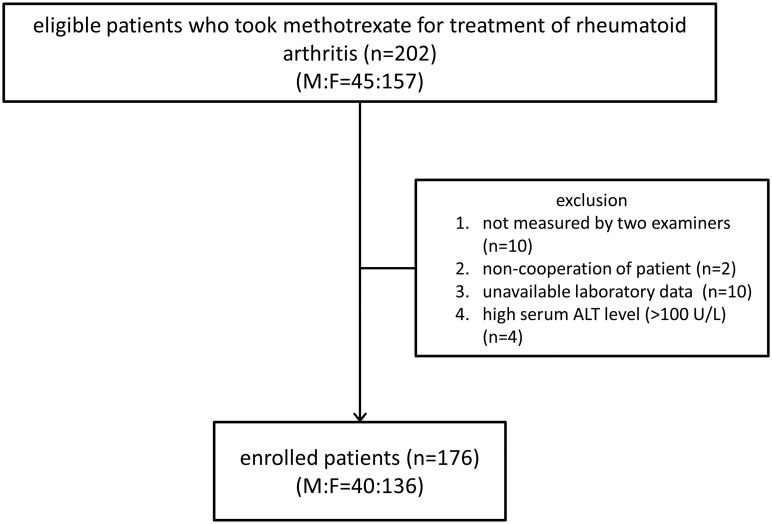
Diagram of subject enrollment.

A total of 202 patients (45 men, 157 women; mean age, 54.0 ± 9.5 years; age range, 29–78 years) who met the inclusion criteria were eligible for this study, but 10 patients were examined by only one examiner due to the physicians’ schedules and LS measurements were not obtained for two patients (1.0% of eligible subjects) due to noncooperation or poor image quality. Among the rest, 14 patients were additionally excluded: laboratory data such as serum cholesterol or ALT levels were not available in 10 patients, and four patients had a serum ALT level over 100 U/L. Therefore, 176 patients (87.1%) were subsequently enrolled (Fig 2). Their demographic, clinical, laboratory, and radiologic characteristics at the time of 2D-SWE examination are listed in Table 1. There were 40 males (22.7%) and 136 females (77.3%) with a mean age of 54.2 ± 9.8 years. Among them, 28.4% (n = 50) were elderly (≥ 60 years old). BMI and waist circumference were investigated in only 168 patients, and the mean waist circumference was 80.8 ± 8.8 cm. The proportion of high BMI (≥ 25 kg/m^2^) was 26.7% (n = 47).

**Table 1:** Demographic, clinical, laboratory, and radiologic characteristics of the enrolled patients.

Characteristics	Study population (n = 176)
Age, years (SD, range)	54.6 (9.3, 31–78)
Sex, male (%)	40 (22.7%)
Body mass index, kg/m^2^ (SD, range)^1^	23.4 (3.0, 16.2–32.0)
Waist circumference, cm (SD, range)^1^	80.8 (8.8, 65–103)
ALT, mg/dL (SD, range)	22.4 (12.6, 6–90)
Cholesterol, mg/dL (SD, range)	189.2 (33.5, 114–286)
SD^M^ (SD, range)	1.61 (1.94, 0.45–18.26)
ROI^M^ (SD, range)	19.19 (2.47, 7.70–25.60)

ALT: alanine transaminase; SD: standard deviation; SD^M^: mean value of standard deviations; ROI^M^: mean diameter of the regions of interest.

^1^n = 168.

### Interobserver reproducibility of 2D-SWE

**Fig 3:**
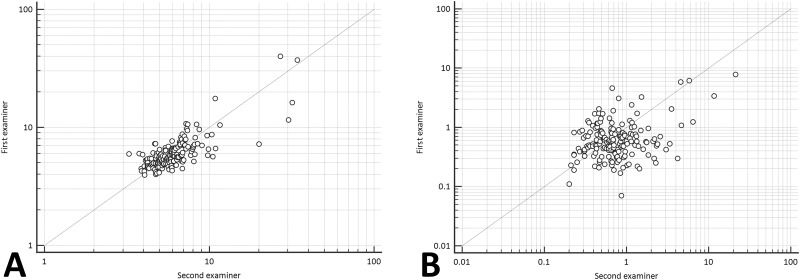
Correlation of liver stiffness (A) and mean value of standard deviation in the region of interest (B) between examiners 1 and 2. The ICC values are 0.783 for liver stiffness and 0.521 for SD^M^, respectively.

The single measurement of ICC (3, 1) was 0.725 (95% confidence interval (CI), 0.647–0.788) for median LS; 0.784 (95% CI, 0.720–0.835) for mean LS; 0.521 (95% CI, 0.404–0.622) for SD^M^ (Fig 3); and 0.242 (95% CI, 0.102–0.374) for ROI^M^, respectively.

### Factors related to interobserver differences in LS

In the univariate linear regression analysis, BMI, waist circumference, SD^M^, ROI^M^, and difference of ROI^M^ were associated with interobserver differences in LS (*p* = 0.020, *p* = 0.019, *p* < 0.001, *p* < 0.001, and *p* = 0.009, respectively). In multivariate linear regression analysis, however, only SD^M^ and ROI^M^ remained independently associated with the interobserver difference of LS (*p* < 0.001 and *p =* 0.021, respectively) (Table 2).

**Table 2:** Univariate and multivariate analyses for interobserver differences in liver stiffness.

	Univariate			Multivariate (R^2^ = 0.254)		
	B value	SD	P value	B value	SD	P value
Age	0.002	0.001	0.156	-0.001	0.001	0.419
Sex	0.016	0.029	0.584			
Cholesterol	0	0	0.61			
ALT	-0.001	0.001	0.395			
Body mass index	0.01	0.004	0.020	-0.005	0.006	0.403
Waist circumference	0.004	0.002	0.019	-0.001	0.002	0.742
SD^M^	0.122	0.019	<0.001	0.132	0.024	<0.001
Difference of SD^M^	0.029	0.034	0.402			
ROI^M^	-0.326	0.076	<0.001	-0.187	0.008	0.021
Difference of ROI^M^	0.222	0.084	0.009	0.071	0.083	0.394

SD: standard deviation; ALT: alanine transaminase; SD^M^: mean value of standard deviations; ROI^M^: mean diameter of the regions of interest.

### Factors related to interobserver discrepancy in LS-based fibrosis stage

**Fig 4:**
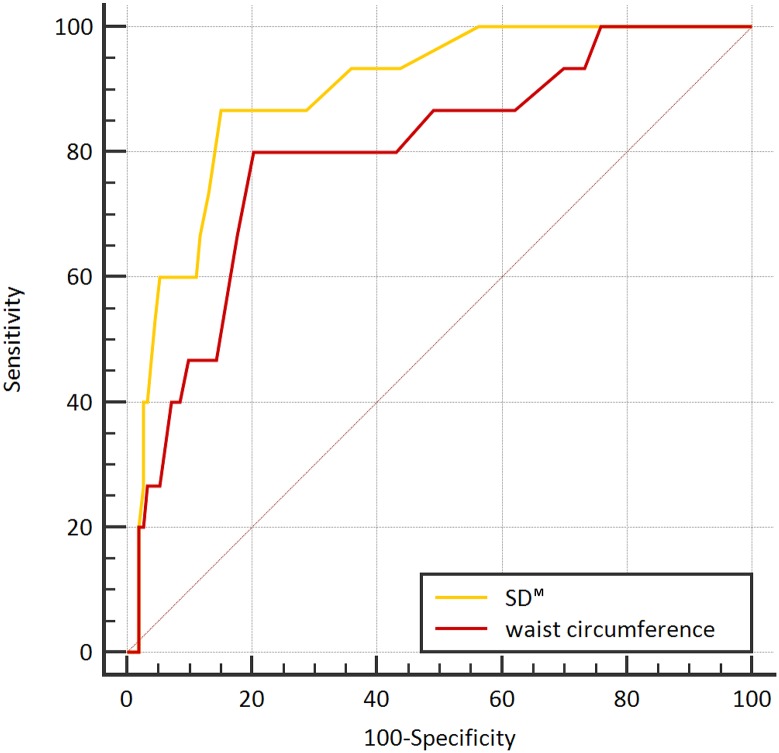
ROC curves for predicting interobserver discrepancies in liver fibrosis determined by 2D-SWE.

Discordance of at least one stage of fibrosis between both measurements was noted in 15 pairs of measurement (8.52%), discordance of at least two stages was noted in six pairs (1.14%), and discordance of three stages was noted in one pair (0.57%) (S1 Table). Significantly related factors of these discrepancies were waist circumference (p = 0.018) and SD^M^ (p = 0.008) in the multivariate logistic regression analysis. ROC curve analysis was carried out to assess the association between interobserver discrepancy in LS-based fibrosis stage and the waist circumference and SD^M^. The corresponding area under the ROC curve (AUC) values were 0.798 (95% CI, 0.729 to 0.856) for waist circumference and 0.894 (95% CI, 0.839 to 0.936) for SD^M^ (Table 3). AUC of SD^M^ was significantly higher than the other variables in predicting interobserver discrepancy in liver fibrosis. The differences between these AUC values of waist circumference and SD^M^ were not statistically significant (p = 0.129). Fig 4 shows the ROC curves for waist circumference and SD^M^ for the diagnosis of interobserver discrepancy in liver fibrosis. The data-driven best cut-off value for SD^M^ was 1.4.

**Table 3:** Receiver operating characteristic curve analyses of related factors for diagnostic accuracy of 2D-SWE in predicting interobserver discrepancy in LS-based fibrosis stage.

	Waist circumference	SD^M^
AUC	0.818	0.894
Standard error	0.0583	0.0374
95% CI	0.752–0.873	0.839–0.936
P value	<0.001	<0.001
Cut-off	>85	>1.4

ROC: receiver operating characteristic; AUC: area under the ROC curve; CI: confidence interval; SD^M^: mean value of standard deviations.

## Discussion

In this prospective study, we found that some of the parameters during measurement could be related to interobserver agreement of LS. These investigation factors, such as SD of ROI or interquartile range of LS measurements, have been used in elastography as a reliability standard [15, 20, 21, 22, 23]. In a study evaluating interobserver concordance in the assessment of liver fibrosis in human immunodeficiency virus/hepatitis C virus-coinfected patients using transient elastography, interquartile range was associated with the absolute values of the difference between two examiners [21]. High BMI and low-grade fibrosis are also related to lower interobserver concordance [22]. Our study also found that BMI and waist circumstance were significant factors in increasing interobserver differences in the univariate analyses, but not in the multivariate analysis. On the other hand, the investigation factors, SD^M^ and ROI^M^, were significant in assessing interobserver differences of LS values by 2D-SWE for both the univariate and multivariate analyses. These investigation factors have merit, in that the examiner can immediately recognize and adjust using them during examination of LS measurement.

SD has been proposed as a reliable criterion before. Recently, Thiele and his colleagues introduced SD and coefficient of variation (SD divided by the numerical mean) as objectively reliable criteria [20]. Because the coefficient of variation is widely used to express the precision and repeatability of a chemical assay, they tested new criteria, including SD and coefficient of variation, to evaluate the precision of the diagnosis of liver cirrhosis and clinically significant portal hypertension (≥ 10 mmHg of hepatic venous pressure gradient) and revealed that measurements with lower SD and larger ROI were most accurate for the diagnosis of both. However, they did not address whether these reliable criteria would also be useful to evaluate the repeatability of measurements such as interobserver agreement, so we dealt with this issue in the present study.

Along with SD^M^, the multivariate analysis indicated that ROI^M^ was also a significant factor that influenced interobserver differences in the measurement of LS. As ROI^M^ increased, interobserver difference of LS decreased. Bilgili et al. also observed an effect of ROI size on interobserver variance [24]. A smaller ROI can make the chances of sampling error increased; on the other hand, a larger ROI is more vulnerable to heterogeneity. However, the examiners generally prefer that the ROI is as large as possible for sufficient sampling. These investigation factors might have some advantages. First, because the SD and ROI diameter are displayed on the monitor along with mean stiffness value as soon as the examiner performs a single measurement, they can help the examiner determine the reliability of the 2D-SWE examination at once, rather than after the end of all measurements (transient elastography requires 10 repetitions to check the reliability of the measurements). Second, they encourage examiners to make a more homogeneous and saturated filling of the color map without any large defects controlling patients’ breathing and changing location of the transducer in the intercostal space. Pellot-Barakat et al. revealed that breathing motion could degrade the data quality of LS including the percent of non-filling in the color map and the LS measurement during an apneic status was superior to that during free breathing [25]. Our data quality was not inferior because we performed LS measurement during an expiratory apneic status.

In ROC curve analysis, we found a significant association between parameters including BMI, waist circumference, and SD^M^ and interobserver discrepancy in LS-based fibrosis stage. Among them, SD^M^ had the largest AUC value, and the best cut-off value for SD^M^ was 1.4 (86.67% sensitivity, 85.09% specificity). As the AUC value of a factor increases, that factor may more precisely predict over- or under-staging. As a result, an SD^M^ higher than 1.4 increased the risk of discordance in LS-based fibrosis stage by 2D-SWE. Patients with BMI higher than 23.8 kg/m^2^ and a waist circumference larger than 84 cm also risked interobserver discrepancy in liver fibrosis. Among them, the SD^M^ was the best parameter, but there was no statistically significant difference based on waist circumference or BMI.

An important caveat in the results of the present study is that these values should be applied in patients with less severe degrees of fibrosis, not cirrhosis, because our patients had a relatively mild form of hepatic fibrosis from long-term administration of methotrexate for the treatment of rheumatoid arthritis. Microscopically, fibrotic structures in cirrhosis are heterogeneous, increasing the SD of a single measurement of elastography. Accordingly, SD would not be applied as a parameter for interobserver variability in cirrhotic patients, and additional study is needed for that setting.

We found that 2D-SWE was a reproducible and noninvasive method for LS measurement. Good interobserver agreement for LS measurement was shown, and most cases (91%) were concordant in classifying LS-based fibrosis stage. Interobserver agreement for SD^M^ was considered fair. Ferraioli et al. [12] assessed intraobserver and interobserver reproducibility of SWE in 42 healthy volunteers without analyzing clinical factors. In contrast, the present study evaluated interobserver agreement of 2D-SWE for screening of hepatic fibrosis in a larger number of patients with long-term medication of the hepatotoxic drug methotrexate.

Nevertheless, our study had several limitations. First, we were unable to consider some factors possibly relating to LS measurement, such as thickness of the subcutaneous fat layer [26]. Second, three radiologists alternated performing 2D-SWE in pairs each day based on random allocation. Because their experience levels were not exactly the same, there could potentially be an interobserver variability depending on the combination differences of the pairs of examiners (e.g. examiner A & B; B & C; C & A).

## Conclusions

Interobserver reproducibility of 2D-SWE in measuring LS in non-cirrhotic liver was good. SD^M^ and ROI^M^ were associated with interobserver differences of LS, and SD^M^ offers a reference factor for the examiner to minimize discordance in LS-based fibrosis stage.

## Supporting information

S1 Table LS-based fibrosis stage by two examiners using cutoff values of liver stiffness with 2D-SWE. Fifteen cases (8.52%) were discordant between two examiners. Cutoff values for LS-based fibrosis stage were referred from [19].
(DOC)

S1 Dataset All laboratory, liver stiffness, standard deviation, and ROI diameters are included. (XLSX)

## Abbreviations

2D-SWE: two-dimensional shear wave elastography
ALT: alanine transaminase
AUC: area under the curve
BMI: body mass index
ICC: intraclass correlation coefficient
LS: liver stiffness
ROC: receiver operating characteristic
ROI: region of interest
ROI^M^: the mean diameter of regions of interest
SD: standard deviation
SD^M^: the mean value of standard deviations
US: ultrasonography
