# Supplementary material for: 2-dimensional shear wave elastography: Interobserver agreement and factors related to interobserver discrepancy
Source: PLoS One. 2017 Apr 17;12(4):e0175747. doi: 10.1371/journal.pone.0175747 (PMC5393601; doi:10.1371/journal.pone.0175747)
Supplement: S1 Table — Fifteen cases (8.52%) were discordant between two examiners. Cutoff values for LS-based fibrosis stage were referred from [19]. (DOC) [file pone.0175747.s001.doc]

**S1 Table. LS-based fibrosis stage by two examiners using cutoff values of liver stiffness with 2D-SWE.** Fifteen cases (8.52%) were discordant between two examiners. Cutoff values for LS-based fibrosis stage were referred from [19].

|  | | Second examiner | | | | |
| --- | --- | --- | --- | --- | --- | --- |
|  |  | F0-F1 | F2 | F3 | F4 | Total |
| First  examiner | F0-F1 | 155 | 4 | 2 | 1 | 162 |
|  | F2 | 3 | 2 | 0 | 0 | 5 |
|  | F3 | 3 | 0 | 1 | 1 | 5 |
|  | F4 | 0 | 0 | 1 | 3 | 4 |
|  | Total | 161 | 6 | 4 | 5 | 176 |
